# Evaluating the impact of social deprivation on Press Ganey® Outpatient Medical Practice Survey Scores

**DOI:** 10.1186/s12955-020-01639-y

**Published:** 2021-06-19

**Authors:** Andrew R. Stephens, Jared W. Potter, Andrew R. Tyser, Nikolas H. Kazmers

**Affiliations:** 1 School of Medicine, University of Utah, 30N 1900E, Salt Lake City, UT 84132 USA; 2 Department of Orthopaedics, University of Utah, 590 Wakara Way, Salt Lake City, UT 84108 USA

**Keywords:** Satisfaction, Social deprivation, Socioeconomic, Access to healthcare

## Abstract

**Background:**

Social deprivation has been shown to affect access to health care services, and influences outcomes for a variety of physical and psychological conditions. However, the impact on patient satisfaction remains less clear. The objective of this study was to determine if social deprivation is an independent predictor of patient satisfaction, as measured by the Press Ganey® Outpatient Medical Practice Survey (PGOMPS).

**Methods:**

We retrospectively reviewed unique new adult patient (≥ 18 years of age) seen at a tertiary academic hospital and rural/urban outreach hospitals/clinics between January 2014 and December 2017. Satisfaction was defined a priori as achieving a score above the 33rd percentile. The 2015 Area Deprivation Index (ADI) was used to determine social deprivation (lower score signifies less social deprivation). Univariate and multivariable binary logistic regression were used to determine the impact of ADI on PGOMPS total and provider sub-scores while controlling for variables previously shown to impact scores (wait time, patient age, sex, race, specialty type, provider type, and insurance status).

**Results:**

Univariate analysis of PGOMPS total scores revealed a 4% decrease in odds of patient satisfaction per decile increase in ADI (*p* < 0.001). Patients within the most deprived quartile were significantly less likely to report satisfaction compared to the least deprived quartile (OR 0.79, *p* < 0.001). Multivariable analysis revealed that the odds of achieving satisfaction decreased 2% for each decile increase in ADI on the Total Score (*p* < 0.001), independent of other variables previously shown to impact scores. For PGOMPS Provider Sub-Score, univariate analysis showed that patients in the lowest ADI quartile were significantly less likely be satisfied, as compared to the least deprived quartile (OR 0.77; 95% CI 0.70–0.86; *p* < 0.001). A 5% decrease in a patient being satisfied was observed for each decile increase in ADI (OR 0.95; 95% CI 0.94–0.96; *p* < 0.001).

**Conclusions:**

Social deprivation was an independent predictor of outpatient visit dissatisfaction, as measured by the Press Ganey® Outpatient Medical Practice Survey. These results necessitate consideration when developing health care delivery policies that serve to minimize inequalities between patients of differing socioeconomic groups.

## Background

Social deprivation incorporates not only socioeconomic status (SES), but also includes a person’s education level, social standing, and access to resources that are available to the general population. The impact of social deprivation on patient health has been well documented. Individuals with greater levels of social deprivation are at an increased risk of major diseases including cardiovascular conditions [1–3], diabetes [4], cirrhosis [5], hypertension [6, 7], and an increased incidence of trauma [8–10]. Similarly, lower SES has been correlated with a worse prognosis and a greater likelihood of disease progression for a variety of conditions, and a higher rate of surgical complications [11–20]. Lower SES also affects access to health care services including preventative screening [21] and surgical or procedural interventions [22–25]. Higher levels of social deprivation are also associated with worse patient-reported functional and psychological outcomes [26–31].

Specific to patient satisfaction, social deprivation has been associated with lower satisfaction on metrics including the Hospital Consumer Assessment of Healthcare Providers and Systems Survey (HCAHPS), the English General Practice Survey, the General Practice Assessment Survey, the National Research Corporation Healthcare Market Guide Survey, and the Primary Care Assessment Tool, and the Kaiser National Household Survey [32–38].

The Press Ganey® Outpatient Medical Practice Survey (PGOMPS) is another commonly used survey utilized to measure patient satisfaction with the process of outpatient care delivery that has been utilized for over 30 years, and is increasingly used by health care systems across the United States. Previous work has demonstrated that PGOMPS is susceptible to a high ceiling rate (29%) similar to findings for other patient satisfaction surveys such as HCAHPS. PGOMPS was also demonstrated to have high internal consistency reliability (0.79–0.96) and convergent validity [39]. Although Nieman et al. [40] demonstrated that lower socioeconomic status correlated with worse PGOMPS scores among the pediatric surgical population, we are unaware of reports evaluating the effects of socioeconomic status on PGOMPS scores across a large multidisciplinary health care system, or literature assessing for an impact of social deprivation on PGOMPS scores.

Our primary study aim was to determine whether social deprivation is associated with patient satisfaction as measured by the PGOMPS Total Score. The secondary aim was to determine the impact of social deprivation on a patient’s satisfaction with their provider, as measured by the PGOMPS Provider Sub-Score. Our null hypotheses are that social deprivation does not impact patient satisfaction scores as measured by the PGOMPS Total Score and the Provider Sub-Score.

## Methods

Our institutional review board approved this retrospective study. Our institution has contracted with Press Ganey Corporation to prospectively collect patient satisfaction scores connected to all outpatient encounters, and evaluates these scores in attempt to improve care delivered. Following each outpatient visit, an e-mail is sent to a patient requesting they complete the PGOMPS. If the survey is incomplete after 5 days, an additional email reminder is sent. Patients may access the survey for up to 30 days post-visit. The Press Ganey Corporation compiles the survey scores, then reports the Total Scores and Provider Sub-Scores back to our institution. Score calculation is performed by the Press Ganey Corporation using proprietary algorithms.

PGOMPS questions are measured on a Likert scale ranging from 1 (indicating very poor) to 5 (indicating very good) and are converted to a 0–100 scale. The survey is comprised of six subdomains: access, moving through your visit, nurse or assistant, care provider, personal issues, and overall assessment for a total of 25 questions and the Total Score is calculated from the mean scores from each of these six individual subdomains using proprietary equations [41]. Similarly, a Provider Sub-Score is computed from the provider-specific questions.

We considered for inclusion all adults (≥ 18 years of age) presenting to a single tertiary academic medical center for a new, outpatient visit seen by physicians, physicians assistants (PA), or nurse practitioners (NP) between January 2014 and December 2017 with a completed Press Ganey Survey, and residence within the state of Utah. Only first new (to the specific provider) patient visits were included, and return and postoperative patient visits were excluded. Patients with incomplete surveys that precluded calculation of a Total Score, a primary language other than English, those lacking a listed address, and those with only a listed post office box, were excluded. Additionally, patients who reported waiting over 6 h to be seen by their provider were excluded as this likely to represent a data entry error. Providers with less than 30 Total Score responses were excluded, given that this small sample size has been stated by the Press Ganey Corporation to lead to inadequate validity [42].

Inclusion also required a 2015 Area Deprivation Index (ADI) value in order to quantify social deprivation for each patient [43]. Therefore, patients with a Post Office Box address were excluded, as a specific ADI cannot be calculated. The ADI value encompasses 17 socioeconomic status factors, including variables such as income, education level, and housing type, that are taken into consideration when assigning ADI scores for a given Zip+4 code, and provides a percentile score on a national basis [44]. Zip+4 codes covers an average of 10–20 homes [45, 46]. Health Resources and Services Administration census data was used in the initial development of ADI and the scores are regularly updated to include the most recent American Community Survey data [46, 47]. ADI has been utilized in several previous studies [30, 31, 44, 46, 48–54], and a higher percentile represents a greater level of social deprivation.

Eligible patient visits were identified by electronic data acquisition of their associated PGOMPS scores and corresponding demographic and visit characteristic data. Wait times in the waiting room and exam room are estimated by the patient after the visit when they complete the survey. The total wait time was calculated as the sum of these two estimates. Satisfaction was defined a priori as receiving a score greater than the 33^rd^ percentile, as per prior studies [41, 55–57].

Continuous variables were summarized as mean ± standard deviation (SD) and categorical variables were summarized as count and percentages (%). The median and interquartile range were calculated for ADI. Potential associations between predictor variables and Total or Provider Sub-Score was identified by univariate binary logistic regression. Separate univariate binary logistic regression models were run with ADI as a continuous variable to predict odds of satisfaction, and categorically by comparing patients in top (scores of 76–100) versus bottom (scores of 0–24) quartiles for ADI on a national level. Provider specialty type was categorized into one of three divisions: internal medicine, surgical, or other. ‘Other’ specialty type was defined as any non-surgical specialty that did not necessitate an internal medicine residency (neurology, dermatology, anesthesiology, etc.). Predictor variables such as patient age, sex, and race, total wait time, specialty type, provider type (physician, nurse, PA, and other), and insurance (commercial, Medicare, Medicaid, workers’ compensation and other government insurance) were analyzed. Multivariable binary logistic regression models with backward stepwise term selection (cutoff α = 0.1) were then used to determine factors associated with Total and Provider Sub-Score satisfaction, as defined by exceeding versus not exceeding the 33^rd^ percentile score, using predictor variables found to be significant in the respective univariate analyses.

## Results

**Fig. 1:**
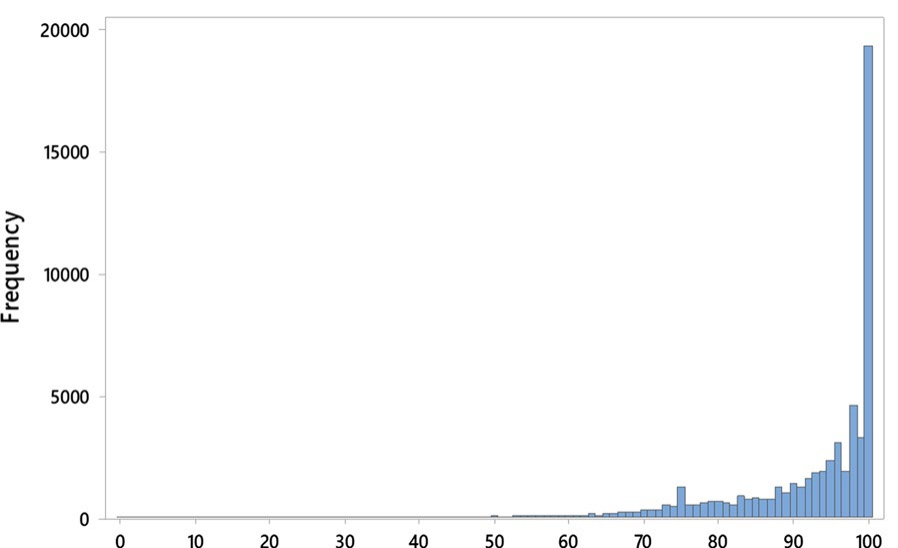
Histogram for the Press Ganey Total Score

**Fig. 2:**
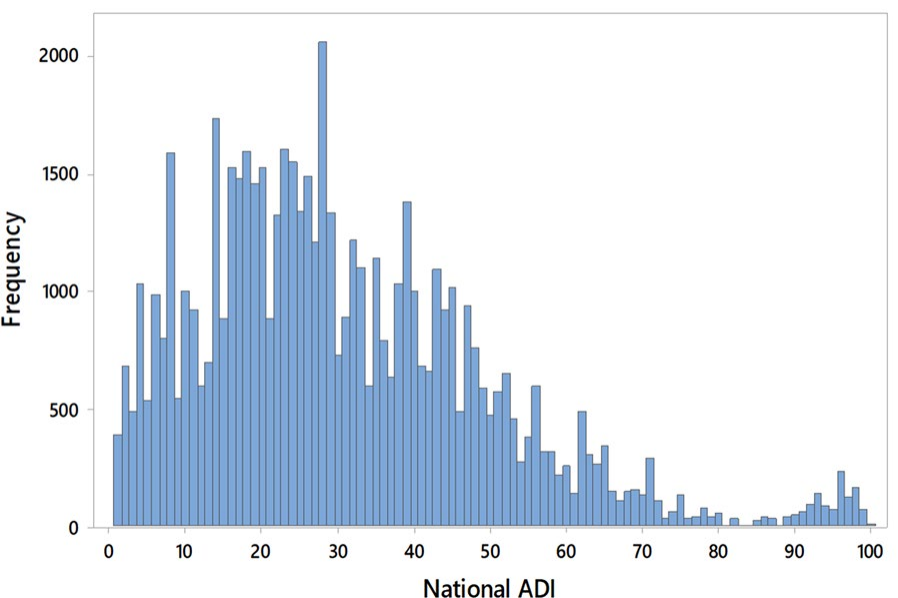
Histogram of Area Deprivation Index values

A total of 61,698 new patient visits with associated PGOMPS Total Scores were identified during our study period. The following exclusions were made: 1117 patients for not having an available ADI, 610 patients for a primary language other than English, and 16 for reported wait times > 6 h. Of the 59,955 included patients, mean age was 51.7 ± 17.1 years, 59.3% were female and 90.5% were White. The PGOMPS Total Score averaged 91.4 ± 12.0 with a 33^rd^ percentile cutoff of 91.7 (Fig. 1). A mean of 93.1 ± 14.8, and 33^rd^ percentile cutoff of 97.5, were observed for the Provider Sub-Score. Mean ADI was 30.8 ± 19.2 (median 28.0; interquartile range 17.0–42.0; range 1–100; Fig. 2). Additional patient demographics and visit data are listed in Table 1.

**Table 1:** Baseline patient characteristics

Factor	Value (n = 59,955)
Demographics	
Age (mean years (SD))	51.7 (17.1)
Area Deprivation Index (National Percentile)	30.8 (19.2)
Race	
White	54,251 (90.5%)
Other	2491 (4.2%)
Asian	1391 (2.3%)
Black or African American	437 (0.7%)
American Indian/Native Alaskan	250 (0.4%)
Native Hawaiian/Pacific Islander	277 (0.5%)
Unknown	858 (1.4%)
Sex (female)	38,330 (63.9%)
Insurance	
Medicaid	943 (1.6%)
Medicare	14,376 (24.1%)
Other government (IHS, VA)	8123 (13.6%)
Commercial	36,033 (60.5%)
Workers' Compensation	137 (0.22%)
Visit characteristics	
Press Ganey Scores	
Total Score	
Mean (SD)	91.4 (11.9)
33rd percentile	91.7
Provider Sub-Score	
Mean (SD)	93.1 (14.8)
33rd percentile	97.5
Specialty type	
Internal medicine	10,552 (17.6%)
Medicine other	26,748 (44.6%)
Surgical	22,655 (37.8%)
Provider type	
Nurse	2944 (4.9%)
Other	3456 (5.8%)
Physician assistant	5654 (9.4%)
Physician	47,878 (79.9%)
Unknown	23 (0.0%)
Wait time (Minutes)	14.0 (16.5)

Continuous data presented as mean ± standard deviation; categorical data presented as number of patients and (percentage)

*HIS* Indian Health Service, *SD* standard deviation, *VA* Veterans Affairs

Univariate analysis showed that patients in the most deprived quartile were significantly less likely be satisfied, as compared to the least deprived quartile, for the PGOMPS Total Score (OR 0.79; 95% CI 0.71–0.87; *p* < 0.001). For each decile increase in ADI, the likelihood of a patient being classified as satisfied decreased by an OR of 0.96 (95% CI 0.95–0.96; *p* < 0.001; Table 2). The PGOMPS Total Score was found to be significantly associated with patient age, sex, race, insurance status, wait time. Provider type and specialty type had no statistically significant correlation with the Total Score (Table 2).

**Table 2:** Univariate analysis for the Press Ganey Total Score

Factor	Odds ratio (OR)		Coefficient	Coefficient standard error	*p* value
	OR	95% confidence interval			
Age*	1.061	(1.055 · 1.066)	0.012	0.0005	< **0.001**
Area Deprivation Index^†^	0.956	(0.948 · 0.965)	− 0.004	0.0004	< **0.001**
Insurance	–	–	–	–	–
Commercial	*Reference category*	–	–	–	–
Medicaid	0.743	(0.652 · 0.847)	− 0.297	0.066	< **0.001**
Medicare	1.341	(1.286 · 1.398)	0.293	0.021	< **0.001**
Other Government Insurance	1.098	(1.044 · 1.155)	0.093	0.026	< **0.001**
Workers' compensation	0.687	(0.490 · 0.963)	− 0.376	0.173	**0.029**
Race	–	–	–	–	**0.001**^‡^
White	*Reference category*	–	–	–	–
Other	0.867	(0.798 · 0.942)	− 0.143	0.042	**0.001**
Asian	0.608	(0.546 · 0.676)	− 0.498	0.055	< **0.001**
Black or African American	0.761	(0.629 · 0.922)	− 0.273	0.098	**0.005**
American Indian and Alaska Native	0.688	(0.535 · 0.884)	− 0.374	0.128	**0.003**
Native Hawaiian and other Pacific Islander	1.162	(0.900 · 1.500)	0.150	0.130	0.25
Sex	–	–	–	–	–
Female	Reference category	–	–	–	–
Male	1.043	(1.001 · 1.080)	0.042	0.018	**0.020**
Provider type	–	–	–	–	< **0.001**^c^
Physician	*Reference category*	–	–	–	–
Nurse	1.018	(0.942 · 1.101)	0.018	0.040	0.65
Other	1.069	(0.994 · 1.150)	0.066	0.037	0.074
Physician Assistant	0.999	(0.943 · 1.059)	− 0.001	0.030	0.98
Specialty type					
Internal medicine	*Reference category*	–	–	–	–
Medicine other	1.001	(0.954 · 1.049)	0.001	0.024	0.99
Surgical	0.960	(0.9143 · 1.008)	− 0.041	0.025	0.097
Wait time^§^	0.766	(0.760 · 0.772)	− 0.053	0.001	< **0.001**

Bolded* p*-values denote statistial significance

*Per 5 years of additional age

^†^Per additional 10 percentile points

^‡^*p* values for the overall univariate binary logistic regression model. Subsequent *p* values listed are for pairwise comparisons

^§^Per additional 5 min

^c^*P*-values for the variable category in the multivariable binary logistic regression model. Subsequent* p*-values listed are for individual comparisons

For the PGOMPS Provider Sub-Score, univariate analysis showed that patients in the lowest ADI quartile were significantly less likely be satisfied, as compared to the least deprived quartile (OR 0.77; 95% CI 0.70–0.86; *p* < 0.001). For each decile increase in ADI, there was a decrease in the likelihood of a patient being satisfied by an OR of 0.95 (95% CI 0.94–0.96; *p* < 0.001; Table 3). Scores also correlated patient age, race, insurance status, wait time, provider type, and specialty type. Patient sex had no statistically significant association with the Provider Sub-Scores (Table 3).

**Table 3:** Univariate analysis for the Press Ganey Provider Sub-Score

Factor	Odds ratio (OR)		Coefficient	Coefficient standard error	*p* value
	OR	95% confidence interval			
Age*	1.052	(1.047 · 1.057)	0.010	0.001	< **0.001**
Area Deprivation Index^†^	0.953	(0.945 · 0.961)	− 0.005	< 0.001	< **0.001**
Insurance	–	–	–	–	–
Commercial	*Reference category*	–	–	–	–
Medicaid	0.798	(0.700 · 0.911)	− 0.225	0.067	**0.001**
Medicare	1.255	(1.204 · 1.308)	0.227	0.021	< **0.001**
Other Government Insurance	1.130	(1.074 · 1.189)	0.122	0.026	< **0.001**
Workers' Compensation	0.829	(0.589 · 1.169)	− 0.187	0.175	0.29
Race	–	–	–	–	**0.001**^‡^
White	*Reference category*	–	–	–	–
Other	0.900	(0.828 · 0.978)	− 0.106	0.043	0.99
Asian	0.706	(0.634 · 0.787)	− 0.348	0.055	< **0.001**
Black or African American	0.783	(0.646 · 0.949)	− 0.245	0.098	**0.013**
American Indian and Alaska Native	0.801	(0.621 · 1.034)	− 0.222	0.130	0.088
Native Hawaiian and Other Pacific Islander	1.274	(0.982 · 1.653)	0.242	0.133	0.069
Sex	–	–	–	–	–
Female	*Reference category*	–	–	–	–
Male	0.978	(0.945 · 1.013)	− 0.022	0.018	0.21
Provider type	–	–	–	–	< **0.001**^c^
Physician	*Reference category*	–	–	–	–
Nurse	1.256	(1.158 · 1.362)	0.228	0.041	< **0.001**
Other	1.280	(1.187 · 1.381)	0.247	0.039	< **0.001**
Physician Assistant	0.970	(0.913 · 1.024)	− 0.034	0.029	0.25
Specialty type					
Internal medicine	*Reference category*	–	–	–	–
Medicine other	0.947	(0.903 · 0.993)	− 0.055	0.024	**0.025**
Surgical	0.886	(0.843 · 0.930)	− 0.121	0.025	< **0.001**
Wait time^§^	0.883	(0.878 · 0.889)	− 0.025	0.001	< **0.001**

Bolded* p*-values denote statistial significance

*Per 5 years of additional age

^†^Per additional 10 percentile points

^‡^*p* values for the overall univariate binary logistic regression model. Subsequent *p* values listed are for pairwise comparisons

^§^Per additional 5 min

^c^*P*-values for the variable category in the multivariable binary logistic regression model. Subsequent* p*-values listed are for individual comparisons

Multivariable analysis revealed a significant negative association between satisfaction on the PGOMPS Total Score and ADI. This was independent of patient age, specialty type, provider type, race, and wait time. Specifically, for each decile increase in ADI the likelihood of achieving satisfaction was decreased by an OR of 0.98 (95% CI 0.97–0.99; *p* < 0.001; Table 4). Multivariable analysis also revealed a significant negative association between the PGOMPS Provider Sub-Score and ADI which was independent of patient age, specialty type, provider type, race, and wait time such that achieving satisfaction decreased by and OR of 0.97 (95% CI 0.96–0.98; *p* < 0.001; Table 5) for each decile increase in ADI.

**Table 4:** Multivariable analysis for the Press Ganey Total Score

Factor*	Odds ratio (OR)		Coefficient	Coefficient standard error	*p* value
	OR	95% confidence interval			
Age^†^	1.063	(1.056 · 1.071)	0.012	0.001	< **0.001**
Area Deprivation Index^c^	0.976	(0.967 · 0.985)	− 0.002	< 0.001	< **0.001**
Insurance	–	–	–	–	–
Commercial	*Reference category*	–	–	–	–
Medicaid	0.901	(0.783 · 1.037)	− 0.104	0.072	0.147
Medicare	1.034	(0.978 · 1.092)	0.033	0.028	0.24
Other Government Insurance	1.132	(1.072 · 1.195)	0.124	0.028	< **0.001**
Workers' compensation	0.821	(0.570 · 1.182)	− 0.197	0.186	0.29
Race	–	–	–	–	**0.001**^‡^
White	*Reference category*	–	–	–	–
Other	1.031	(0.943 · 1.127)	0.031	0.046	0.50
Asian	0.669	(0.597 · 0.750)	− 0.402	0.058	< **0.001**
Black or African American	0.938	(0.763 · 1.153)	− 0.064	0.105	0.54
American Indian and Alaska Native	0.794	(0.607 · 1.039)	− 0.231	0.137	0.093
Native Hawaiian and Other Pacific Islander	1.274	(1.064 · 1.838)	0.335	0.139	**0.016**
Sex	–	–	–	–	–
Female	Reference category	–	–	–	–
Male	0.956	(0.921 · 0.994)	− 0.045	0.019	0.022
Provider type	–	–	–	–	< **0.001**^§^
Physician	*Reference category*	–	–	–	–
Nurse	0.973	(0.896 · 1.056)	− 0.028	0.042	0.51
Other	0.853	(0.789 · 0.921)	− 0.159	0.039	< **0.001**
Physician assistant	1.023	(0.962 · 1.088)	0.022	0.031	0.48
Wait time^||^	0.764	(0.878 · 0.889)	− 0.054	0.001	< **0.001**

Bolded* p*-values denote statistial significance

*Specialty type was included as a predictor in the model, but was insignificant based on a backward step-wise term selection threshold of α = 0.10

^†^Per 5 years of additional age

^‡^Per additional 10 percentile points

^§^*p* values for the overall univariate binary logistic regression model. Subsequent *p* values listed are for pairwise comparisons

^||^Per additional 5 min

^c^*P*-values for the variable category in the multivariable binary logistic regression model. Subsequent* p*-values listed are for individual comparisons

**Table 5:** Multivariable analysis for the Press Ganey Provider Sub-Score

Factor*	Odds ratio (OR)		Coefficient	Coefficient standard error	*p* value
	OR	95% Confidence Interval			
Age^†^	1.061	(1.054 · 1.068)	0.012	0.001	< **0.001**
Area Deprivation Index^c^	0.967	(0.958 · 0.976)	− 0.003	< 0.001	< **0.001**
Insurance	–	–	–	–	–
Commercial	*Reference Category*	–	–	–	–
Medicaid	0.927	(0.809 · 1.062)	− 0.076	0.069	0.27
Medicare	0.973	(0.922 · 1.026)	− 0.028	0.027	0.30
Other Government Insurance	1.156	(1.097 · 1.219)	0.145	0.027	< **0.001**
Workers' Compensation	1.000	(0.703 · 1.423)	< − 0.001	0.180	1.000
Race	–	–	–	–	**0.001**^‡^
White	*Reference Category*	–	–	–	–
Other	1.025	(0.940 · 1.117)	0.025	0.044	0.58
Asian	0.767	(0.686 · 0.857)	− 0.266	0.057	< **0.001**
Black or African American	0.911	(0.747 · 1.112)	− 0.093	0.102	0.36
American Indian and Alaska Native	0.889	(0.684 · 1.155)	− 0.118	0.134	0.38
Native Hawaiian and Other Pacific Islander	1.469	(1.125 · 1.919)	0.385	0.136	**0.005**
Sex	–	–	–	–	–
Female	Reference Category	–	–	–	–
Male	0.917	(0.884 · 0.952)	− 0.087	0.019	< **0.001**
Provider type	–	–	–	–	< **0.001**^§^
Physician	*Reference Category*	–	–	–	–
Nurse	1.218	(1.120 · 1.324)	0.197	0.043	< **0.001**
Other	1.264	(1.166 · 1.370)	0.234	0.041	< **0.001**
Physician Assistant	0.977	(0.920 · 1.037)	− 0.023	0.031	0.44
Wait time^||^	0.886	(0.881 · 0.891)	− 0.024	0.001	< **0.001**

Bolded* p*-values denote statistial significance

*Specialty type was included as a predictor in the model, but was insignificant based on a backward step-wise term selection threshold of α = 0.10

^†^Per 5 years of additional age

^‡^Per additional 10 percentile points

^§^*p* values for the overall univariate binary logistic regression model. Subsequent *p* values listed are for pairwise comparisons

^||^Per additional 5 min

^c^*P*-values for the variable category in the multivariable binary logistic regression model. Subsequent* p*-values listed are for individual comparisons

## Discussion

The main finding of this study was that increased social deprivation was associated with decreased outpatient satisfaction, as measured by the Press Ganey Outpatient Medical Practice Survey. This observation was observed for both the Press Ganey Total Score and the Provider Sub-Score, and was independent of several factors previously shown to have a large magnitude of impact on patient satisfaction including wait time, patient age, sex and race [41, 55, 57, 58]. The secondary findings of the study are in-line with much prior work on patient satisfaction and associated factors such as patient age, sex, race, insurance status, wait time, provider type, and specialty type [41, 55, 57, 58].

The importance of understanding how socioeconomic factors affect the utilization of and access to the healthcare system is becoming increasingly evident. The role of a patient’s social and economic circumstances in their overall physical and mental health has been well-elucidated [3, 11, 31, 46, 48, 53, 58–60], and our findings are consistent with a limited number of previous studies in documenting an association between socioeconomic status and patient satisfaction scores.

Young et al. [61] demonstrated that average income levels based on zip codes and lower patient satisfaction scores are correlated among elderly patients seen in various specialty clinics. McFarland et al. [38] evaluated 934,000 patients and showed Hospital Consumer Assessment of Healthcare Providers and Systems (HCAHPS) survey scores were directly correlated with education level, which has been used as a surrogate for socioeconomic status in the literature. Additionally, Nieman et al. [40] demonstrated that lower socioeconomic status correlated with worse PGOMPS scores among the pediatric surgical population.

Critically, this study does not address whether the quality of health care differed based on a patient’s social deprivation. The PGOMPS does not measure health care quality, but rather satisfaction with the process of outpatient health care provision. It is important to make the distinction between quality and satisfaction, particularly when interpreting evidence suggesting that social deprivation may influence a patient’s perception of the care they received [22, 62–65]. Along those lines, this study does not address the underlying causes of differences in patient satisfaction scores between patients with different levels of social deprivation. Arpey et al. [62] demonstrated that patients of lower SES were more likely to perceive their economic status as influencing their care than those of higher socioeconomic brackets. Verlinde et al. conducted a systematic review evaluating how SES affects physician patient communication. In their study they found that patients of lower SES are less likely to participate in shared decision making and ask questions [66]. Schroder et al. [67] pointed out that patients in the bottom socioeconomic quartile had less medical knowledge and were less likely to desire to play an active role in their disease management than patients in the highest quartile. Studies by Wright et al. [30] and Okoroafor et al. [31] demonstrated that patients were more likely to report high levels of anxiety and depression in worse social deprivation indexes, and Tyser et al. [58] and Tisano et al. [68] independently showed that patients with worse PROMIS anxiety and depression scores were less likely to report satisfaction on the PGOMPS. An additional study by Schroder et al. [69] found that patients of lower SES were more likely to wait to seek care for their heart disease until after they suffered a myocardial infarction. Previous literature has also shown that physicians who treat patients with greater disease severity and worse prognosis are more likely to receive lower patient satisfaction scores [70–73].

Objective discrepancies of care based on socioeconomic status have, however, been documented. Govindarajan and Schull found that patients residing in economically deprived neighborhoods were less likely to have advanced paramedic teams dispatched to their location and had greater transport time to hospitals when controlled for distance than those residing in less economically deprived neighborhoods [24]. Patel et al. [23] found that time between initial encounter for an ACL tear to surgery was greater for pediatric patients from lower socio-economic settings. The systematic review conducted by Verlinde et al. [66] also found that lower SES patients received less overall communication and fewer explanations directed to their understanding level than those of higher SES. It remains uncertain if these inequalities of care are a result of limited access of care and insurance/payment difficulties, or rather due to inherent biases. Clearly, the interplay between socioeconomic status, social deprivation, and the healthcare delivery process is complex. Further work is needed to evaluate for and potentially reduce discrepancies of care that these patients may experience in line with the overall goal of providing equitable and high-quality care.

The Patient Protection and Affordable Care Act enables Medicare to make incentive payments to hospitals based on specific quality domains that include the patient experience of care, and have in turn been used to adjust physician compensation [74, 75]. Our findings may also help inform health care policy makers and/or administrators in decision making surrounding attaching patient satisfaction scores to various methods of reimbursements. The impact of such policies should be evident: without accounting for the impact of a patient’s economic disadvantage on satisfaction scores, providers who have reimbursement tied to satisfaction scores may be disincentivized from caring for patients with, or working in areas with, high levels social deprivation. This could further perpetuate the disparities that these policies are attempting to correct. An example of this was demonstrated in a study that evaluated the impact of the 2019 peer group stratification of Medicare’s Hospital Readmission Reduction Program (HRRP) in the United States. The HRRP allows for a penalization to be enacted if hospitals have readmission rates greater than 30 days [76]. In 2016, The United States Congress passed the twenty-first Century Cures Act allowing HRRP to take into consideration the effect of social deprivation on readmission rates [77]. Under HRRP in 2019, hospital performance was stratified into quintiles based on patient socioeconomic status and the proportion enrolled in Medicare and Medicaid. The cost of readmission penalties to hospitals and subsequently physician reimbursements were cut in half for hospital’s in the most deprived quintile as demonstrated by Joynt Maddox et al. [78]. The importance of accounting for SES in evaluation of health care quality has also been demonstrated outside of the United States [79].

There are several limitations of this study. The generalization of our findings to other health care systems with differing regional and patient demographics is limited given that our study was conducted at a single institution treating a population that is predominately white. Furthermore, our institution provides care for patients from a large geographical distribution. Many patients, often from underserved and economically disadvantaged areas, travel up to several hours to be seen by specialists at our institution. The expectations, and therefore satisfaction, of these patients be different from other hospital systems with smaller catchment areas. Our study is also limited by a non-response bias, which is also an inherent limitation of the PGOMPS in general. Previous literature from our institution has shown the PGOMPS response rate to ranges from 8.9 to 16.5% [39, 41]. Tyser et al. [56] found that responders differed from non-responders in terms of age, sex, and insurance type. These factors are a real-world limitation of PGOMPS, and should not only be taken into account when interpreting study results, but also when determining the applicability of the survey as a determinant of vale of care and reimbursement rates. Although we only included new patient visit patient encounters, it is possible that a patient’s economic situation, and satisfaction with care, could potentially change throughout a treatment course. Lastly, the magnitude the association between ADI and satisfaction are seemingly small in comparison to patient age and wait time, but the effects are additive for increasing deciles of social deprivation and the comparison between highest and lowest quartiles demonstrates a significant difference.

## Conclusion

Increased social deprivation is a predictor of lower patient satisfaction, as measured by the Press Ganey Outpatient Medical Practice Survey, and this effect is independent of other known factors that have a large impact on scores. These results necessitate consideration in order to develop health care delivery policies that serve to minimize inequalities between patients of differing socioeconomic groups.

## Data Availability

We do not feel that it is appropriate to publish the raw data used to perform this study, as it is largely proprietary and administered by a private corporation. The data can be available from the authors upon request.

## Appendix

See Table 6.

**Table 6:** Multivariable analysis for the Press Ganey Total Score Forward Selection

Factor*	Odds ratio (OR)		Coefficient	Coefficient standard error	*p* value
	OR	95% confidence interval			
Age^†^	1.013	(1.011 · 1.014)	0.012	0.001	< **0.001**
Area deprivation Index^c^	0.999	(0.998 · 1.000)	− 0.001	< 0.001	**0.008**
Insurance	–	–	–	–	–
Commercial	*Reference category*	–	–	–	–
Medicaid	1.324	(1.142 · 1.536)	0.281	0.075	< **0.001**
Medicare	1.078	(1.022 · 1.137)	0.075	0.027	**0.006**
Other Government Insurance	1.137	(1.077 · 1.201)	0.129	0.028	< **0.001**
Workers' Compensation	0.786	(0.514 · 1.201)	− 0.241	0.216	0.265
Race	–	–	–	–	**0.001**^‡^
White	*Reference category*	–	–	–	–
Other	1.106	(1.025 · 1.193)	0.100	0.039	**0.009**
Asian	0.765	(0.672 · 0.871)	− 0.267	0.066	< **0.001**
Black or African American	1.012	(0.813 · 1.260)	0.012	0.112	0.915
American Indian and Alaska Native	0.803	(0.603 · 1.096)	− 0.207	0.153	0.174
Native Hawaiian and Other Pacific Islander	1.612	(1.249 · 2.081)	0.477	0.130	< **0.001**
Provider type	–	–	–	–	< **0.001**^§^
Physician	*Reference category*	–	–	–	–
Nurse	1.048	(0.964 · 1.140)	0.047	0.043	0.271
Other	1.069	(0.988 · 1.156)	0.067	0.040	0.095
Physician Assistant	1.169	(1.098 · 1.244)	0.156	0.031	< **0.001**
Wait time^||^	0.937	(0.935 · 0.939)	− 0.066	0.001	< **0.001**
Specialty type	–	–	–	–	–
Internal medicine	*Reference category*	–	–	–	–
Medicine other	0.932	(0.885 · 0.981)	− 0.070	0.026	**0.008**
Surgical	1.002	(0.950 · 1.057)	0.002	0.027	0.934

Bolded *p*-values denote statistial significance

*Sex was included as a predictor in the model, but was insignificant based on forward step-wise term selection threshold of α = 0.10

^†^Per 5 years of additional age

^‡^Per additional 10 percentile points

^§^*p* values for the overall univariate binary logistic regression model. Subsequent *p* values listed are for pairwise comparisons

^||^Per additional 5 min

^c^*p*-values for the variable category in the multivariable binary logistic regression model. Subsequent *p*-values listed are for individual comparisons

## Abbreviations

SES: Socioeconomic 283 status
HCAHPS: Hospital Consumer Assessment of Healthcare Providers and Systems Survey
PGOMPS: Press Ganey® Outpatient Medical Practice Survey
NP: Nurse practicioner
PA: Physician assistant
ADI: Area Deprivation Index
